# Image quality and diagnostic value of diffusion-weighted breast magnetic resonance imaging: Comparison of acquired and computed images

**DOI:** 10.1371/journal.pone.0247379

**Published:** 2021-02-22

**Authors:** Hye Shin Ahn, Sung Hun Kim, Ji Youn Kim, Chang Suk Park, Robert Grimm, Yohan Son

**Affiliations:** 1 Department of Radiology, Chung-Ang University Hospital, Chung-Ang University College of Medicine, Seoul, Republic of Korea; 2 Department of Radiology, College of Medicine, Seoul St. Mary’s Hospital, The Catholic University of Korea, Seoul, Republic of Korea; 3 Department of Radiology, College of Medicine, Yeouido St. Mary’s Hospital, The Catholic University of Korea, Seoul, Republic of Korea; 4 Department of Radiology, College of Medicine, Incheon St. Mary’s Hospital, The Catholic University of Korea, Icheon, Republic of Korea; 5 MR Applications Development, Siemens Healthcare, Erlangen, Germany; 6 Siemens Healthineers Ltd., Seoul, Republic of Korea; Medical University of Vienna, AUSTRIA

## Abstract

**Purpose:**

To compare the image quality of acquired diffusion-weighted imaging (DWI) and computed DWI and evaluate the lesion detectability and likelihood of malignancy in these datasets.

**Materials and methods:**

This prospective study was approved by our institutional review board. A total of 29 women (mean age, 43.5 years) underwent DWI between August 2018 and April 2019 for 32 breast cancers and 16 benign breast lesions. Three radiologists independently reviewed the acquired DWI with b-values of 1000 and 2000 s/mm^2^ (A-b1000 and A-b2000) and the computed DWI with a b-value of 2000 s/mm^2^ (C-b2000). Image quality was scored and compared between the three DWI datasets. Lesion detectability was recorded, and the lesion’s likelihood for malignancy was scored using a five-point scale.

**Results:**

The A-b1000 images were superior to the A-b2000 and C-b2000 images in chest distinction, fat suppression, and overall image quality. The A-b2000 and C-b2000 images showed comparable scores for all image quality parameters. C-b2000 showed the highest values for lesion detection among all readers, although there was no statistical difference in sensitivity, specificity, positive predictive value, negative predictive value, and accuracy between the DWI datasets. The malignancy scores of the DWI images were not significantly different among the three readers.

**Conclusions:**

A-b1000 DWI is suitable for breast lesion evaluations, considering its better image quality and comparable diagnostic values compared to that of A-b2000 and C-b2000 images. The additional use of computed high b-value DWI may have the potential to increase the detectability of breast masses.

## Introduction

Dynamic contrast-enhanced magnetic resonance imaging (DCE-MRI) is currently the most sensitive method for breast cancer detection [1–4]. Although DCE-MRI is generally used for breast cancer patients, it has limitations such as the use of a contrast agent, gadolinium. Gadolinium is widely known for its side effect of nephrogenic systemic fibrosis in patients with reduced renal function. Gadolinium is also retained in other parts of the body including the brain. Its long-term effects are uncertain [5]. Therefore, the use of gadolinium-based contrast agents should be minimized. The potential role of non-enhanced MRI using diffusion-weighted imaging (DWI) combined with other sequences in breast cancer patients has been proposed in several studies [6–9].

DWI is a functional MR technique that provides information based on the microscopic movement of water molecules in tissues and allows an indirect assessment of tissue microstructures and cellularity [10]. The derived apparent diffusion coefficient (ADC) values can differentiate between malignant and benign breast lesions. Therefore, it has been shown to improve the diagnostic specificity and positive predictive value (PPV) of dynamic contrast-enhanced (DCE)-MRI [11, 12]. A high b-value (>1000 s/mm^2^) DWI is usefulfor lesion detection and characterization as it maximizes the tissue contrast between pathologic and normal tissues [13–16]. However, high b-value DWI can be limited by a low signal-to-noise ratio (SNR) due to the longer echo times required and artifacts such as eddy current-induced distortions.

Computed DWI is a mathematical technique that calculates any b-value image from the acquired DWI data with at least two different b-values [17]. Computed b-value images do not have the same disadvantages as acquired DWI because they are not directly acquired but are calculated from acquired b-value images in a voxel-wise manner. Several previous studies have reported that computed high b-values are useful for lesion detection in patients with breast cancer [18–20]. In particular, Bickel et al. [20] compared DWIs with different synthetic b-values between b1000 and b2000 s/mm^2^ and concluded that synthetically increased b-values may improve image quality and lesion visibility in breast DWI. Although the aforementioned references reported on the usefulness of computed high b-values for lesion detection in breast cancer patients [18–20]; there are no published study comparing both acquired and computed DWI with high b-values of 1000 and 2000 s/mm^2^ yet. Therefore, the purpose of this study was to compare the image quality of acquired DWI (with b-values of 1000 s/mm^2^ and 2000 s/mm^2^) and computed DWI (with b-value 2000 s/mm^2^) and evaluate the lesion detectability and likelihood of malignancy of the three datasets.

## Material and methods

### Study population

This prospective study was approved by the Catholic Medical Center Office of Human Research Protection Program (CMC-OHRP) and Institutional Review Board (Approval No. KC16EISI0542). Informed consent was obtained from all the patients. Between August 2018 and April 2019, 32 consecutive women who underwent breast MRI to confirm breast lesions (24 patients with biopsy-proven invasive cancers and eight patients with biopsy-proven benign breast masses) were recruited. Among these patients, three were excluded from the study (consent withdrawal in one case, and referral to other hospitals in two cases). Thus, a total of 52 breast lesions identified in 29 female patients (age range, 21–65 years; mean age, 43.52 ± 9.39 years) were included.

### Image acquisition

MRI was performed using a 3-T MR scanner (MAGNETOM Verio; Siemens Healthcare, Erlangen, Germany) with a dedicated breast coil in the prone position. All DWI images were acquired before the administration of a contrast agent. For DWI, the readout-segmented echo-planar imaging (EPI) sequence was applied using the following parameters: TR/TE, 6300/68 ms; FOV, 320 × 160 mm; matrix size, 192 × 96; fat saturation, an acquisition time of 6 min 32 s; and automatically generated ADC maps. Imaging was performed with b-values of 0, 1000 and 2000 s/mm^2^. The acquired diffusion-weighted images were exported and post-processed using a prototype software (MR Body Diffusion Toolbox v1.3.0, Siemens Healthcare, Erlangen, Germany). The ADC maps and computed diffusion-weighted images with high b-value (2000 s/mm^2^) were generated from b = 0 and b = 1000 by fitting signal intensities to the Stejskal-Tanner equation S(b) = S0*exp(-b·ADC) with a pixel intensity of S0 in b = 0 s/mm^2^.

### Image analysis

Mammography, ultrasound images, breast MRI (T2-weighted images, dynamic contrast-enhanced MRI, and DWI) and pathologic reports were reviewed by one radiologist (S.H.K, the supervisor) with 15 years of experience in breast imaging who did not participate in the DWI analysis. A total of 52 breast lesions (36 malignant and 16 benign) were identified in 29 patients. The mean tumor size was 24.1 mm (range, 5–87 mm). All malignant lesions (n = 36) were pathologically proven to be invasive ductal carcinomas (n = 33), invasive lobular carcinoma (n = 1), and ductal carcinoma in situ (n = 2), including index cancers, daughter nodules, and contralateral cancer. Among a total of 16 benign lesions, eight were pathologically confirmed benign masses (five fibroadenomas, two benign phyllodes tumors, and one fibrocystic change) and the other eight were diagnosed as benign at follow-up (all lesions were stable on after more than two years of follow-up). Imaging in 29 patients with three different DWI sets (A-b1000, A-b2000, and C-b2000) resulted in 87 individual datasets that were provided in random order to the three readers. Three radiologists with 20, 10, and 8 years of experience in breast imaging independently reviewed the images (C.S.P., J.Y.K., and H.S.A.). The ADC maps and b = 0 images were included in the image sets. The readers could check the ADC value of any lesions, but T2-weighted or contrast-enhanced MR images were not provided. First, the readers recorded the image quality factors for each DWI, and then the detectability and likelihood of malignancy of the lesions were evaluated. The image quality factors are presented in Table 1. For image quality, the authors rated each anatomical structure using a score of 0 (non-distinction of anatomy) or 1 (distinction of anatomy). For lesion detectability, the readers were asked to report all distinguishable lesions along with the location (left/right) and image number containing the detected lesion. They were also asked to assign the likelihood of malignancy scored using a 5-point scale (1 = definitely not malignant, 2 = probably not malignant, 3 = indeterminate, 4 = probably malignant, and 5 = definitely malignant). The radiologists knew that all the patients had a breast mass but were blinded to the final diagnosis and the number of masses, laterality, and quadrant location of the lesion(s). Finally, the supervisor reviewed the lesions detected by the three radiologists and matched the detected lesions with the 52 previously identified reference lesions.

**Table 1:** Criteria for comparison of image quality in DWI images.

Characteristics
Distinction of anatomical structure
0–1: skin, parenchyma-fat, chest wall, sternum
Homogeneity (Right vs. Left)
1: Right > Left
2: Right = Left
3: Right < Left
Ghosting artifacts
1: Definitely confounding interpretation
2: Present, but no impact on interpretation
3: No artifact
Fat suppression
1: No suppression
2: Inhomogeneous fat suppression
3: Homogeneous fat suppression
Background noise
1: Definitely confounding interpretation
2: Present, but no impact on interpretation
3: No artifact
Overall image quality
1: Poor
2: Moderate
3: Good
4: Excellent

DWI = diffusion-weighted imaging

### Statistical analysis

Regarding the image quality, the inter-reader agreement was assessed by the intraclass correlation coefficient (ICC) [21]. An ICC greater than 0.75 was considered excellent agreement (ICC < 0.4, poor; and ICC 0.4–0.75, fair-to-good). The average scores of the three readers were calculated and used for the analysis. Differences in image quality between the DWI datasets were compared using the Chi-square test or Fisher’s exact test. For the detection and likelihood of the lesions, the predictive performance for malignancy (a likelihood score of 3, 4, or 5) was determined by the area under the receiver operating characteristic curve (AUC) analysis, including sensitivity, specificity, PPV, negative predictive value (NPV), and accuracy. All statistical analyses were performed using SAS (version 9.4, SAS Institute Inc., Cary, NC, USA). P-values < 0.05 were considered statistically significant.

## Results

### Image quality among DWI datasets

**Fig 1:**
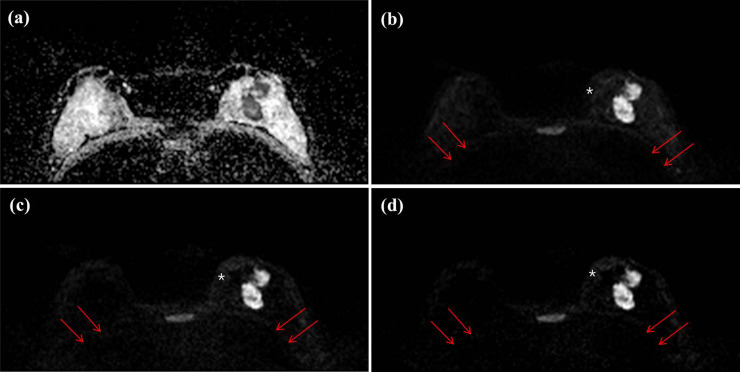
Axial DWIs of a 47-year-old woman with invasive ductal carcinoma (IDC, 4.2 cm and histologic grade II) in her left breast for (a) an ADC image, (b) an acquired b value of 1000 s/mm^2^ (A-b1000) (c) an acquired b value of 2000 s/mm^2^ (A-b2000) and (d) a computed b value of 2000 s/mm^2^ (C-b2000). All of three readers score as 0 (no distinction) for chest wall in C-b2000 image, however chest wall is relatively well demarcated in A-b1000 image (arrows). Subcutaneous fat layer is well suppressed in A-b1000 image, however inhomogeneously suppressed in A-b2000 and C-b2000 image (white asterisks).

Table 2 displays the image quality factors of the pooled data from the three readers and the inter-reader agreement. Among the four factors used to distinguish the anatomical structures, such as skin-line, parenchyma, chest, and sternum, the score for chest wall distinction was significantly lower in A-b2000 and C-b2000 than in A-b1000 (p < 0.001). In the subgroup analysis, there were significant differences between A-b1000 and A-b2000 and between A-b1000 and C-b2000 (p < 0.001 in both), but there was no statistical difference between A-b2000 and C-b2000 (p > 0.990). Similarly, C-b2000 showed significantly lower scores for fat suppression and overall image quality (p < 0.001). However, there was no significant difference between A-b2000 and C-b2000 in fat suppression (p = 0.090) and overall image quality (p = 0.089) (Fig 1). Other parameters, including three anatomical distinctions (skin-line, parenchyma, and sternum), homogeneity, and background noise were not significantly different between the DWI images. The three readers were in fair-to-good inter-reader agreement regarding the overall image quality of the A-b1000 (ICC = 0.705, range 0.453–0.852) images. However, there was poor agreement for A-b2000 (ICC = 0.258, range -0.375–0.852) and C-b2000 (ICC = 0.279, range -0.336–0.638).

**Table 2:** Image quality of DWI datasets and inter-reader agreement.

Parameter	Score range	A-b1000		A-b2000		C-b2000		*p* value
		Mean ± SD	ICC (range)	Mean ± SD	ICC (range)	Mean ± SD	ICC (range)	
Anatomical structure distinction								
Skinline	0–1	0.93 ± 0.26	0.851 (0.725–0.925)	0.97 ± 0.18	0.574 (0.210–0.786)	0.94 ± 0.23	0.421 ((-)0.072–0.709)	0.696
Parenchyma	0–1	0.90 ± 0.31	0.509 (0.089–0.753)	0.90 ± 0.31	0.655 (0.362–0.827)	0.87 ± 0.33	0.379 ((-)0.150–0.688)	0.856
Chest	0–1	0.97 ± 0.18	0.596 (0.251–0.797)	0.66 ± 0.48	0.373 ((-)0.162–0.685)	0.64 ± 0.48	0.695 (0.438–0.847)	<0.001
								<0.001*
								<0.001+
								>0.990§
Sternum	0–1	0.93 ± 0.26	1.000 (1.000–1.000)	0.93 ± 0.26	1.000 (1.000–1.000)	0.97 ± 0.18	1.000 (1.000–1.000)	0.572
Homogeneity	1–3	2.07 ± 0.26	0.321 ((-)0.257–0.659)	2.07 ± 0.26	(-)0.196 ((-)1.215–0.399)	2.08 ± 0.41	(-)0.644 ((-)2.047–0.174)	0.052
Ghosting artifact	1–3	2.53 ± 0.61	0.869 (0.757–0.934)	2.53 ± 0.61	0.780 (0.592–0.889)	2.57 ± 0.60	0.813 (0.653–0.906)	0.846
Fat suppression	1–3	2.48 ± 0.59	0.561 (0.187–0.779)	1.98 ± 0.43	0.197 ((-)0.488–0.596)	1.84 ± 0.40	0.452 ((-)0.016–0.724)	<0.001
								<0.001*
								<0.001+
								0.090§
Background noise	1–3	2.41 ± 0.50	0.331 ((-)0.239–0.664)	2.31 ± 0.54	0.269 ((-)0.355–0.633)	2.30 ± 0.49	0.354 ((-)0.196–0.675)	0.258
Overall image quality	1–4	2.90 ± 0.95	0.705 (0.453–0.852)	2.22 ± 0.77	0.258 ((-)0.375–0.627)	1.98 ± 0.68	0.279 ((-)0.336–0.638)	<0.001
								<0.001*
								<0.001+
								0.089§

Note.—Numbers shows mean values ± standard deviation except for score range and p-value

DWI = diffusion-weighted imaging, ICC = intraclass correlation coefficient, SD = standard deviation

*p-values between A-b1000 and A-b2000

+*p*-values between A-b1000 and C-b2000

§*p*-values between A-b2000 and C-b2000

### Detectability and likelihood of malignancy of the lesions among DWI datasets

**Fig 2:**
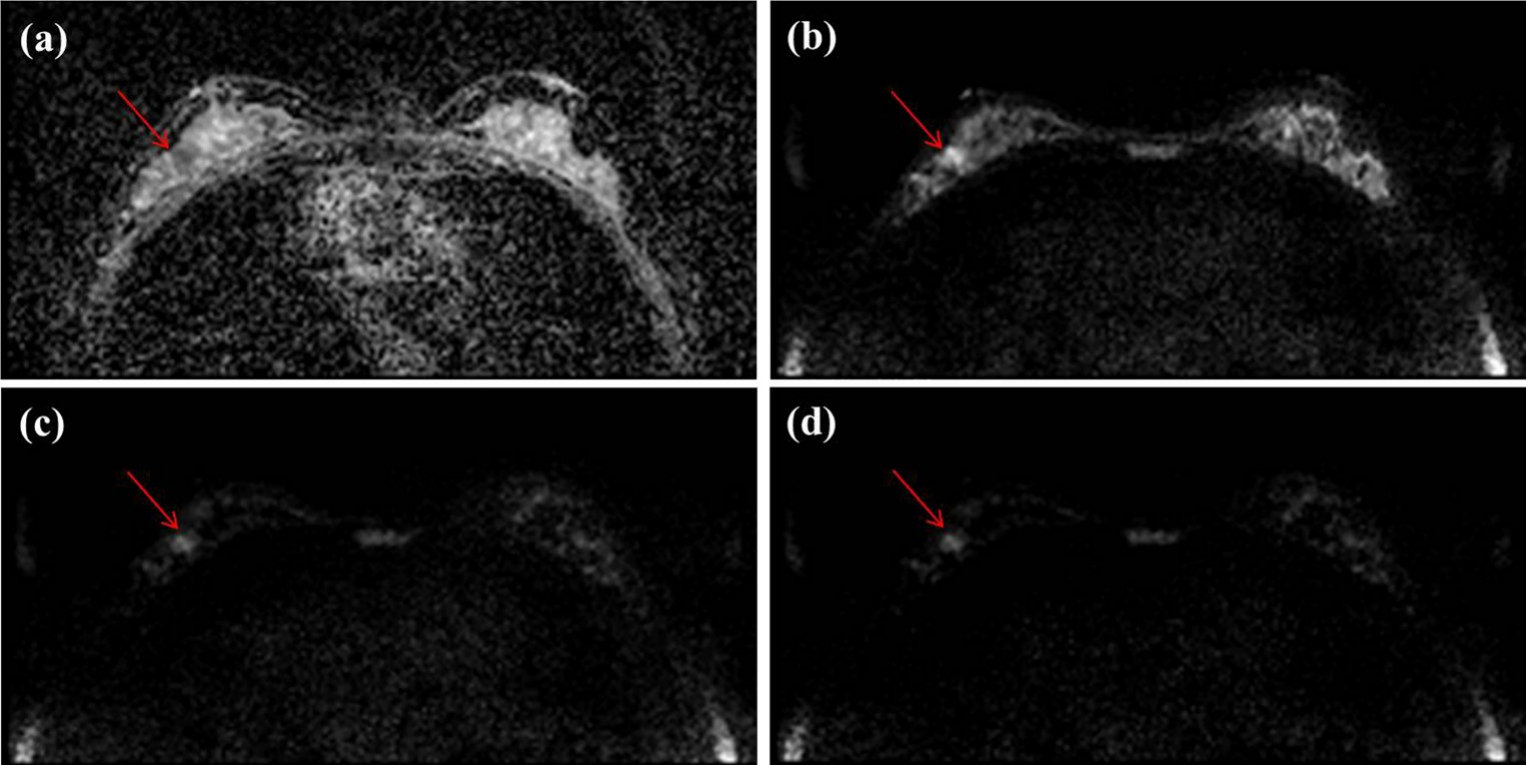
Axial DWIs of a 44-year-old woman with ductal carcinoma in situ (DCIS, 1 cm and histologic grade I) for (a) an ADC image, (b) an acquired b value of 1000 s/mm^2^ (A-b1000) (c) an acquired b value of 2000 s/mm^2^ (A-b2000) and (d) a computed b value of 2000 s/mm^2^ (C-b2000) in her right breast, which was missed by two of three readers in A-b1000, but was well seen in C-b2000. All readers detected this lesion in C-b2000 and two readers rated as 5 and the other rated 3 in malignancy score.

Table 3 summarizes the detection and malignancy scores of the readers for the lesions on the DWI images. For lesion detection, the C-b2000 images showed the highest values for sensitivity, specificity, PPV, NPV, and accuracy compared to the A-b1000 and A-b2000 images and the pooled data. However, there was no significant difference between DWI datasets (Fig 2). In terms of the malignancy scores for the lesions, the three DWI datasets showed variable diagnostic values. The sensitivity and PPV were similar for the three datasets. The mean sensitivity was 97.6% for A-b1000, 94.5% for A-b2000, and 95.8% for the C-b2000 images (p = 0.593). The mean PPV was 84.5% in A-b1000, 81.9% in A-b2000, and 84.3% in C-b2000 images (p = 0.854). The specificity and NPVs tended to be higher in the A-b1000 images than in other image sets, but inter-reader value variations were not statistically significant. The mean specificity was 48.3% for A-b1000, 34.5% for A-b2000, and 29.2% for the C-b2000 images (p = 0.325). The mean NPV was 87.5% in A-b1000, 66.7% in A-b2000, and 63.6% in the C-b2000 images (p = 0.281). The accuracy was higher in the C-b2000 image (78.2%) than in the other images (73.7% in both), but there was no significant difference between the DWI datasets (p = 0.570).

**Table 3:** Lesion detection and likelihood of malignancy of DWI datasets.

	Reader 1	Reader 2	Reader 3	Pooled
Detection				
Sensitivity, %				
A-b1000	88.9 (73.9–96.9)	69.4 (51.9–83.7)	75.0 (57.8–87.9)	77.8 (68.8–85.2)
A-b2000	91.7 (77.5–98.3)	77.8 (60.9–89.9)	83.3 (67.2–93.6)	84.3 (76.0–90.6)
C-b2000	94.4 (81.3–99.3)	80.6 (64.0–91.8)	88.9 (73.9–96.9)	88.0 (80.3–93.4)
*P*-value	0.907	0.518	0.297	0.127
Specificity, %				
A-b1000	37.5 (15.2–64.6)	37.5 (15.2–64.6)	43.8 (19.8–70.1)	39.6 (25.8–54.7)
A-b2000	37.5 (15.2–64.6)	43.8 (19.8–70.1)	37.5 (15.2–64.6)	39.6 (25.8–54.7)
C-b2000	43.8 (19.8–70.1)	50.0 (24.7–75.4)	56.3 (29.9–80.3)	50.0 (35.2–64.8)
*P*-value	0.917	0.776	0.556	0.493
PPV, %				
A-b1000	76.2 (60.6–88.0)	71.4 (53.7–85.4)	75.0 (57.8–87.9)	74.3 (65.3–82.1)
A-b2000	76.7 (61.4–88.2)	75.7 (58.8–88.2)	75.0 (58.8–87.3)	75.8 (67.2–83.2)
C-b2000	79.1 (64.0–90.0)	78.4 (61.8–90.2)	82.1 (66.5–92.5)	79.8 (71.5–86.6)
*P*-value	0.945	0.790	0.693	0.590
NPV, %				
A-b1000	60.0 (26.2–87.8)	35.3 (14.2–61.7)	43.8 (19.8–70.1)	44.2 (29.1–60.1)
A-b2000	66.7 (29.9–92.5)	46.7 (21.3–73.4)	50.0 (21.1–78.9)	52.8 (35.5–69.6)
C-b2000	77.8 (40.0–97.2)	53.3 (26.6–78.7)	69.2 (38.6–90.9)	64.9 (47.5–79.8)
*P*-value	0.877	0.582	0.375	0.180
Accuracy, %				
A-b1000	73.1 (59.0–84.4)	59.6 (45.1–73.0)	65.4 (50.9–78.0)	66.0 (58.0–73.4)
A-b2000	75.0 (61.1–86.0)	67.3 (52.9–79.7)	69.2 (54.9–81.3)	70.5 (62.7–77.5)
C-b2000	78.9 (65.3–88.9)	71.2 (56.9–82.9)	78.9 (65.3–88.9)	76.3 (68.8–82.7)
*P*-value	0.784	0.449	0.296	0.135
**Malignancy score**				
Sensitivity, %				
A-b1000	96.9 (83.8–99.9)	96.0 (79.7–99.9)	100 (87.2–100)	97.6 (91.7–99.7)
A-b2000	97.0 (84.2–99.9)	96.4 (81.7–99.9)	90.0 (73.5–97.9)	94.5 (87.6–98.2)
C-b2000	94.1 (80.3–99.3)	96.6 (82.2–99.9)	96.9 (83.8–99.9)	95.8 (89.6–98.8)
*P*-value	>0.999	>0.999	0.206	0.593
Specificity, %				
A-b1000	40.0 (12.2–73.8)	70.0 (34.8–93.3)	33.3 (7.5–70.1)	48.3 (29.5–67.5)
A-b2000	50.0 (18.7–81.3)	33.3 (7.5–70.1)	20.0 (2.5–55.6)	34.5 (17.9–54.3)
C-b2000	33.3 (7.5–70.1)	50.0 (15.7–84.3)	0.0 (0.0–0.0)	29.2 (12.6–51.1)
*P*-value	0.893	0.311	0.313	0.325
PPV, %				
A-b1000	83.8 (71.9–95.7)	88.9 (77.0–100)	81.8 (64.5–93.0)	84.5 (75.8–91.1)
A-b2000	86.5 (75.5–97.5)	81.8 (64.5–93.0)	77.1 (59.9–90.0)	81.9 (73.2–88.7)
C-b2000	84.2 (68.8–94.0)	87.5 (71.0–96.5)	81.6 (65.7–92.3)	84.3 (76.0–90.6)
*P*-value	0.941	0.752	0.858	0.854
NPV, %				
A-b1000	80.0 (28.4–99.5)	87.5 (47.4–99.7)	100 (29.2–100)	87.5 (61.7–98.5)
A-b2000	83.3 (35.9–99.6)	75.0 (19.4–99.4)	40.0 (5.3–85.3)	66.7 (38.4–88.2)
C-b2000	60.0 (14.7–94.7)	80.0 (28.4–99.5)	100 (100–100)	63.6 (30.8–89.1)
*P*-value	0.794	>0.999	0.167	0.281
Accuracy, %				
A-b1000	78.9 (65.3–88.9)	71.2 (56.9–82.9)	71.2 (56.9–82.9)	73.7 (66.1–80.4)
A-b2000	82.7 (69.7–91.8)	71.2 (56.9–82.9)	67.3 (52.9–79.7)	73.7 (66.1–80.4)
C-b2000	80.8 (67.5–90.4)	76.9 (63.2–87.5)	76.9 (63.2–87.5)	78.2 (70.9–84.4)
*P*-value	0.884	0.746	0.548	0.570

Note.—Data are presented as percentages, with confidence intervals in parentheses.

DWI = diffusion-weighted imaging, PPV = positive predictive value, NPV = negative predictive value

## Discussion

The goal of this study was to compare acquired DWI at b = 1000 and b = 2000 s/mm^2^ and computed DWI at b = 2000 s/mm^2^ in terms of image quality and lesion detectability and likelihood of malignancy for breast lesions. The A-b1000 images were superior to the A-b2000 and C-b2000 images in visualizing chest distinction and fat suppression, as well as the overall image quality. C-b2000 showed the highest values for lesion detection by all readers, although there was no statistical difference in sensitivity, specificity, PPV, NPV, and accuracy between the three DWI datasets. In addition, the malignancy scores of the DWI images showed variable diagnostic values without significant differences among the three readers.

Our hypotheses for image quality are as follows: First, the lower b-value DWI showed better image quality than the higher b-value DWI. Second, the computed high b-value DWI was superior in image quality to the acquired high b-value image. Since a high b-value DWI has inherently low SNR and is sensitive to artifacts, it leads to long acquisition times and degraded image quality. Breast tissues are susceptible to differences between tissue and air interfaces and lung/cardiac motion movements. The lower chest distinction, fat suppression, and overall image quality scores for the high b-value images in the current study may be due to these reasons. The results were comparable to our hypothesis (Fig 1). In this study, we acquired DWI with b-values of 0, 1000 and 2000 s/mm^2^ with an acquisition time of 6 min 32 s. However, acquisition time is shorter in acquired DWI with b-values of 0 and 1000 s/mm^2^ in the same vendor (4 min 27 s). Another hypothesis was that computed DWI showed better image quality than acquired DWI when the same b-value was applied. Previous studies have reported that a computed high b-value DWI is feasible with good SNR, and results in less image distortion [17, 18, 22–24]. This can result in improved lesion detection in patients with malignancies in variable organs. Blackledge et al. [17] reported that computed high b-value DWI improves SNR, diagnostic sensitivity, and specificity for tumor detection in a relatively small and heterogeneous group of patients with different types of cancer. In our study, C-b2000 showed similar values in image quality parameters as the A-b2000 images (Fig 2). O’Flynn et al. [18] compared the image quality of acquired DWI (b = 1150 s/mm^2^) and computed DWI (b = 1500 and b = 2000 s/mm^2^) and reported that the computed DW-MR images produced better image quality according to the mean score of the readers. However, in their study, readers 1 and 2 showed different results. Reader 1 scored computed DWI with b1500 and b2000 significantly higher. However, reader 2 scored both computed DWI image sets marginally lower than the acquired DWI with b1150. The authors suggested that the reason for variation in scoring between the two readers was subjectivity in the qualitative evaluation of the computed DWI, personal preference for image quality, and a learning curve effect associated with reading standard and synthetic images. Our unexpected results including lower ICC values between the readers for high b-value images, may be explained by the above factors. In a future study, we may consider improving the reviewer’s learning curve or consensus status of computed DWI to overcome the lower ICC in the current study.

It is well known that increasing the b-value during image acquisition can increase breast lesion contrast due to higher diffusivity in normal fibroglandular tissue (FGT) versus malignancy [25, 26]. As the b-value increases, the signal intensity of the normal FGT decreases at a rate faster than that of the tumor, making a malignancy more conspicuous. Furthermore, computed images have the advantage of decreasing image distortions due to susceptibility effects and eddy currents, increased SNR, and shortened scan times compared to acquired images with the same high b-value [27]. Therefore, the authors of this study anticipated that C-b2000 would show the highest performance in lesion detection and malignancy scoring among the three DWI datasets. Although C-b2000 showed the highest diagnostic values in lesion detection, there was no statistical difference between the DWI datasets. A recent study by DelPriore et al. [28] compared the conspicuity of breast cancer on computed and acquired high b-value DWI using 3-T MRI and found that the lesion contrast-to-noise ratio (CNR) was higher on the acquired images and began to decrease at b-values greater than 1500 s/mm^2^. However, lesion visibility was not significantly different between acquired and computed images. A study by Tamura et al. [29] suggested improving the conspicuity of breast tumors on computed high b-value DWI by 1.5-T MRI, and the CNR of computed DWI generated from high SNR images and a high number of excitations (NEX) was superior to that of acquired DWI. However, the CNR on computed DWI obtained using the same scanning parameters was inferior to the acquired DWI. In this study, we acquired DWI images with b-values of 0, 1000, and 2000 s/mm^2^, and obtained a computed image with a b-value of 2000 s/mm^2^ generated from DWI with b-values of 0 and 1000 s/mm^2^ with the same scanning parameters, including NEX. This could cause a decrease in the SNR in computed DWI images and affect lesion detection and malignancy scores.

This study had several limitations. First, the small sample size in this study may have affected the statistical significance of the results, such as the diagnostic values of lesion detection. Second, the supervisor settled distinguishable lesions on DWI images by readers as reference standard. The readers did not have enhanced dynamic or T2-weighted images for reference and made comparisons between the DWI images. Therefore, the sensitivity of lesion detection may be overestimated compared to the results of previous studies [18, 19], which could affect the diagnostic values, such as PPV and accuracy. However, the purpose of this study was to compare the image quality and diagnostic values of DWI image sets. The result suggests differences in these points. Third, the likelihood of malignancy was scored using a 5-point scale by the three readers. Bath and Mansson [30] initially demonstrated visual grading characteristics (VGC) analysis for image quality evaluation between two compared modalities. Although VGC may have its intrinsic subjectivity, this evaluation method has been used by many researchers for assessing the diagnostic performance of breast MRI and the results have suggested consistent results between compared modalities [18, 19, 31].

In conclusion, the image quality of acquired DWI at b = 1000 s/mm^2^ was superior to that of acquired DWI at b = 2000 s/mm^2^ and computed DWI at b = 2000 s/mm^2^. Computed DWI at b = 2000 s/mm^2^ showed the highest values for lesion detection, although the difference was not statistically significant. Therefore, acquired DWI at b = 1000 s/mm^2^ can be recommended in 3-T MRI. However, computed DWI at b = 2000 s/mm^2^ may be considered an additional DWI protocol. Further studies in larger cohorts are needed to test the feasibility of high b-value DWI for increasing diagnostic performance in clinical practice.

## List of abbreviations

ADC: Apparent diffusion coefficient
DCE: Dynamic contrast-enhanced
DWI: Diffusion-weighted imaging
MRI: Magnetic resonance imaging
